# Ovarian stem cells: absence of evidence is not evidence of absence

**DOI:** 10.1186/1757-2215-6-65

**Published:** 2013-09-17

**Authors:** Deepa Bhartiya, Kalpana Sriraman, Seema Parte, Hiren Patel

**Affiliations:** 1 Stem Cell Biology Department, National Institute for Research in Reproductive Health, Mumbai 400012, India

**Keywords:** Stem cells, Ovary, Cysts, Lineage tracing, VSELs

## Abstract

**Background:**

Lei and Spradling in a recent study published in PNAS failed to detect ‘germline cysts’ by elegant studies using lineage tracing approach and thus concluded that adult mouse ovaries lack stem cells. They proposed that primordial follicle pool generated during fetal life is sufficient to sustain oogenesis and that there is no renewal of oocytes during adult life. Contrary to their results, we have reported presence of very small pluripotent, embryonic-like stem cells (VSELs), their immediate descendants (OGSCs) and germ cell ‘cysts’ or ‘nests’ (formed by rapid cell division and incomplete cytokinesis) in surface epithelial cell smears of adult sheep, monkey and human ovaries.

**Methods:**

In the present study, ovaries were collected from adult mouse (treated with 5 IU pregnant mare serum gonadotropin, PMSG) and sheep (from slaughter house) and testis from mouse treated with busulphan (25 mg/Kg). Ovarian surface epithelial (OSE) cells and testicular smears were studied for the presence of cysts. Sheep OSE smears were also used to show cytoplasmic continuity amongst the cyst cells studied by immunolocalization and confocal microscopy of stem cells specific markers OCT-4 and SSEA-4.

**Results:**

Cysts were observed and confocal microscopy imaging confirmed cytoplasmic continuity amongst the cells comprising the cysts.

**Conclusions:**

Cysts represent self-renewal and clonal expansion of stem cells with incomplete cytokinesis and are a hallmark feature of stem cells. We suggest the use of PMSG stimulated mouse ovaries and use of more primitive markers like OCT-4 or STELLA rather than MVH for lineage tracing studies to conclusively show presence of stem cells by lineage-tracing studies.

## Introduction

Sphere forming ability is considered a hallmark property of stem cells (cardiosphere, neurosphere, prostatesphere etc.) including cancer stem cells (mammosphere, melanospheres). The early embryo is indeed a sphere and embryonic stem cells also form sphere-shaped embryoid bodies. Similarly germ cell ‘nests’ or ‘cysts’ (germ cells that divide rapidly with incomplete cytokinesis) are well documented in the fetal ovaries [1,2] and chains of clonally expanded stem cells exist in the testes [3]. These structures possess multipotent properties and essentially represent self-renewal and clonal expansion of stem cells with incomplete cytokinesis.

Presence of mammalian ovarian stem cells is a highly debated area of reproductive biology since 2004, when Prof Jonathan Tilly from Harvard Medical School first challenged the long held central doctrine of fixed germ cell pool in females [4]. Although compelling evidence has been provided by several groups in support of postnatal oogenesis, the subject has been challenged and negated by many including Lei & Spradling [1] who found no evidence of stem cells in mouse ovary using genetic recombination approach.

Evidence is emerging that various adult body organs possess two types of stem cells including dormant and active stem cells [5,6]. Similarly two populations of stem cells including relatively quiescent very small embryonic-like stem cells (VSELs) and their actively dividing, immediate descendants ‘progenitors’ termed ovarian germ stem cells (OGSCs) have been reported by our group in mouse, rabbit, sheep, monkey and human ovary surface epithelium (OSE) [7]. The stem cells at times are visualized as small clusters which are the germ cell ‘nests’ or ‘cysts’ representing self-renewal and clonal expansion. Twenty-one days long culture of OSE cells comprising these stem cells result in their spontaneous differentiation into oocyte-like structures [7]. Further we have shown augmentation in process of neo-oogenesis and primordial follicle (PF) assembly in adult mice treated with PMSG [8]. Ovarian cortical tissue cultures to study PF growth have also demonstrated stimulation of stem cells in presence of FSH and bFGF [9]. Further stem cells in sheep OSE undergo clonal expansion and form cysts in response to FSH treatment and that this process is mediated through R3 isoform of FSH receptor [10]. In this brief report we show that just like our earlier published data in rabbit, sheep, monkey and human ovaries, cysts are visualized in adult sheep and mouse ovaries and are more in number in PMSG treated mice.

## Material and methods

For isolation of mouse OSE, normal and PMSG treated (5 IU subcutaneous) ovaries were subjected to enzymatic digestion using a protocol similar to one reported earlier by Symonds et al. [11]. PMSG was injected during di-estrous phase of the estrus cycle and ovaries were collected after 24 hours. Briefly the ovaries were separated from the surrounding bursal tissue and carefully suspended in DMEM containing high glucose (Life Technologies). Each ovary was incubated at 37°C for 30 mins in 0.1 ml of the DMEM containing 0.5 mg/ml of Collagenase Type IV (Life Technologies). Tubes were briefly vortexed for 2 mins and the enzyme reaction was terminated by adding DMEM media supplemented with 15% fetal bovine serum (Life Technologies) and vortexed again for 2 mins after removal of ovaries. Smears were made from dissociated OSE cells on poly-l-lysine coated slides and fixed with 4% paraformaldehyde (PFA) followed by Haematoxylin and Eosin (H & E) staining. Sheep OSE cells were obtained by mechanical scraping of sheep ovaries [7] and smears fixed in 4% PFA and stained with H & E prior to viewing under an upright microscope (Nikon 90i, Japan). Busulphan treated mouse testis was used as a positive control to show the presence of stem cells and cyst-like structures. Briefly the mice were treated with 25 mg/Kg busulphan (Sigma) by intra-peritoneal route and after one month, the chemo-ablated mice testes were used for making smears. The smears were H & E stained and viewed under a microscope to capture representative images.

Germ cell markers OCT-4 and SSEA-4 were immunolocalized on sheep OSE smears and viewed under confocal microscope (LSM 510-META; ZEISS, Germany). Briefly, the cell smears were permeabilized with 0.3% Triton X-100 for 5 mins at room temperature for cytoplasmic marker (OCT-4) whereas this step was omitted for cell surface specific marker (SSEA-4). Non-specific epitopes were blocked by incubation with 10% serum of species specific to secondary antibody used in immuno-staining for one hour at room temperature. Cells were incubated overnight with respective primary antibody at 4°C and subsequently washed with washing buffer (DPBS containing 0.5% BSA and 0.1 mM EDTA) and later incubated with Alexafluor 488 (Molecular Probes, Invitrogen) labeled anti- rabbit IgG (1:1000) or anti-mouse IgG in wash buffer for 1 hour. First smears were washed with washing buffer several times, followed by counter staining for 30 seconds with propidium iodide (0.5 ug/ml) or DAPI (1.47 μM) (both from Sigma-Aldrich). Negative controls were also prepared by omission of primary antibody. Images of representative areas were captured by laser scanning confocal microscope at 63× magnification with further optical zoom using argon laser at λ = 488 nm, blue diode laser at λ = 405 and DPSS laser at λ = 561 nm for FITC, DAPI and PI staining channels respectively.

## Results and Discussion

We have published several papers on gonadal VSELs [7-10,12-14] and have shown that similar to the VSELs in the bone marrow and cord blood [15,16], the VSELs in gonads are spherical cells, smaller than red blood cells, with high nucleo-cytoplasmic ratio, stain very dark with Haematoxylin, do not stain easily with DAPI, express pluripotent (OCT-4A, SSEA-4, Nanog, Sox-2, Rex), primordial germ cells (STELLA, FRAGILIS), VSELs (SCA-1, CD133) and germ cells (VASA, DAZL) specific markers. They are located in the ovary surface epithelium and in the basal epithelium of testicular seminiferous tubules. They are quiescent in nature and self-renew to give rise to the progenitors which divide rapidly with incomplete cytokinesis and occasionally appear as cysts and differentiate further and undergo meiosis to give rise to the gametes.

**Figure 1:**
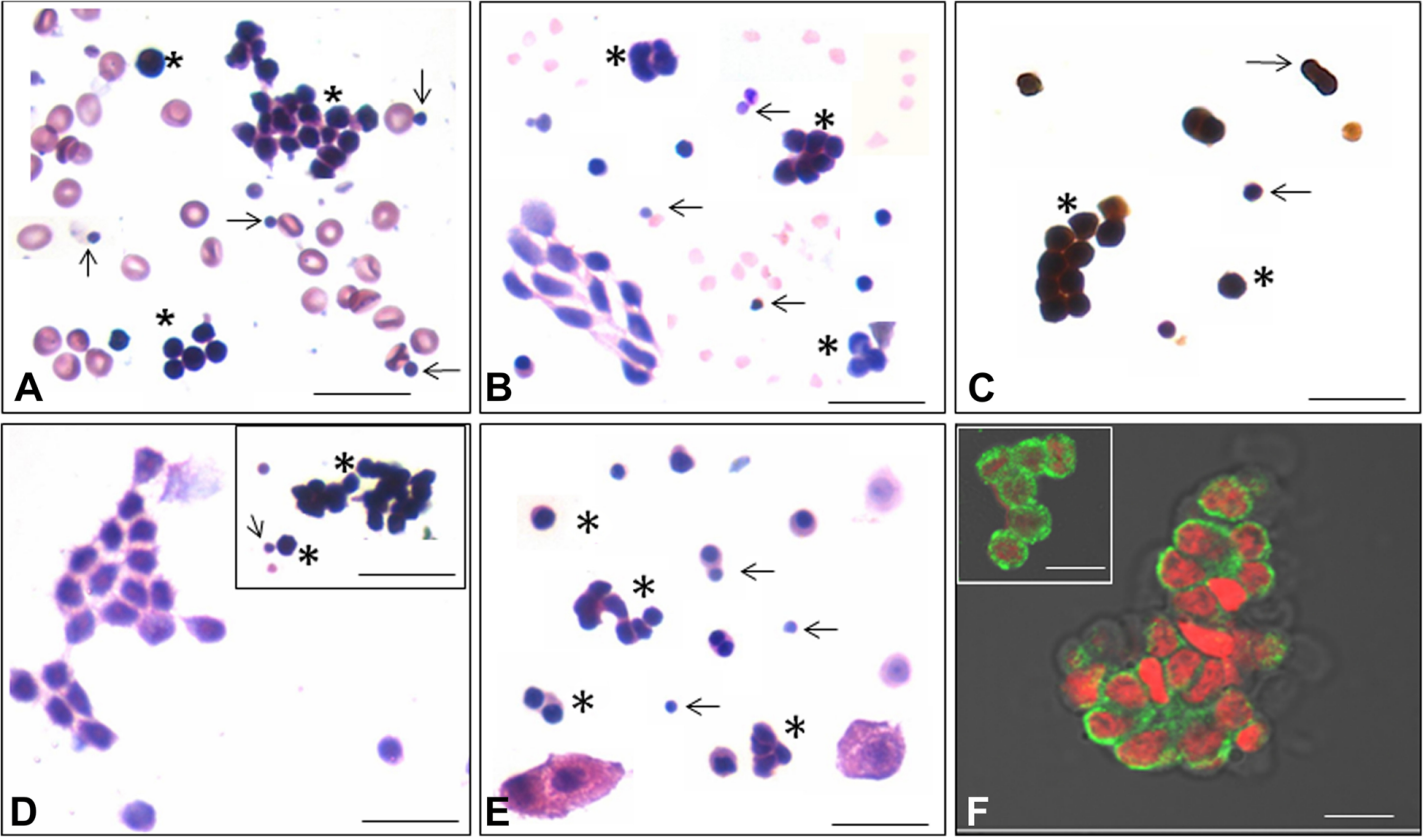
**Analysis of human, sheep and mouse ovarian surface epithelial and mouse testicular cell smears: Haematoxylin and Eosin stained smears of human (A), sheep (B) and mice (D) ovary surface epithelial (OSE) cell smears.** Human OSE smear image is from our earlier publication [14]. Cysts implying stem cell clusters are clearly evident in ovaries of all species. In mice, number of cysts was markedly increased after PMSG treatment **(D** inset**)** compared to normal ovary **(D)**. Immunostaining with anti-PCNA antibody of sheep OSE smears shows that the cysts are positive for PCNA **(C)** suggesting their proliferative state. H & E stained smears of busulphan (25 mg/Kg) treated testis **(E)**. Due to the treatment, all germ cells are completely depleted however; large Sertoli cells with abundant cytoplasm and small, spherical stem cells are clearly visualized in the smears **(E)**. The somatic epithelial cells in ovary **(A**, **B**, **D)** and Sertoli cells in testicular **(E)** smears have abundant cytoplasm and pale stained nuclei whereas the stem cells are spherical in shape, with high nucleo-cytoplasmic ratio and are easily visualized by H & E staining as two distinct populations comprising smaller VSELs and slightly larger progenitors which are ovary germ stem cells (OGSCs) in the ovary and spermatogonial stem cells (SSCs) in the testis. The smaller cells marked by arrow in A-D indicate VSELs whereas the dividing slightly bigger progenitors including cysts are marked by asterisks. Confocal imaging of OCT-4 **(F** inset**)** and SSEA-4 **(F)** in the cysts in sheep OSE smears clearly showing presence of cytoplasmic continuity amongst the dividing stem cells. A-E represents composites prepared by capturing 4-5 fields to show the stem cells and somatic cells together in a small field. Stem cells are not very frequent as shown here. Scale bar represents 20 μm **(A**-**E)** and 10 μm **(F)**.

Three distinct cell types are observed in OSE smears including epithelial cells, red blood cells and stem cells Figure 1D. The epithelial cells are present singly or as sheets with abundant cytoplasm and pale stained nuclei. RBCs are easily observed as biconcave disc-like cells with absent nucleus. The stem cells are spherical with high nucleo-cytoplasmic ratio and dark stained nuclei. Cell clusters or cysts are observed occasionally. Ovarian stem cells and cysts become prominent in mouse OSE smears after PMSG treatment Figure 1D inset. Busulphan treated testis clearly showed the presence of Sertoli cells and two populations of stem cells and cysts Figure 1E Confocal z-stack analysis of cytoplasmic OCT-4 and cell surface SSEA-4 in sheep ovarian cysts showed cytoplasmic continuity amongst the cells comprising the cysts due to rapid proliferation and incomplete cytokinesis. Presence of non follicular cell aggregates expressing germ/stem cell markers that represents cysts in adult ovary has also been demonstrated earlier by others using OCT-4-EGFP transgenic mouse model [17].

However, using mouse VASA homolog (MVH) as a marker and lineage labeling approach, Lei & Spradling [1] did not detect mitotically active germ cell progenitors and cysts, although mouse testis and fetal ovary used as positive controls showed the presence of cysts suggesting that their technique was perfect. Their method was based on detecting actively dividing cells, which would get lineage marked to express YFP through controlled use of tamoxifen, although the expression is not restricted only to the germ cells. As germ cell cysts are actively dividing, they are expected to get lineage marked and express YFP. But they used normal adult mouse ovaries for their study and based on our results and earlier reports [7,8], they may have got positive results had they used mouse ovary after PMSG treatment. Neo-oogenesis in adult ovary is a very subtle process and thus has remained elusive for decades, compared to testes or fetal ovary where the order of magnitude of the same process is very high. A large number of sperm are produced in the testes and oocytes in fetal ovary compared to few oocytes per estrus cycle during adult life. At the dose of tamoxifen, which was used to control the number of cells expressing YFP by Lei and Spradling [1] it is quite possible that the subtle process of cyst formation in adult ovary remained undetected, even though it could be detected in testes and fetal ovary.

Interestingly, in the Lei and Spradling study the number of germ cell cysts that were lineage marked even in fetal ovary appear to be very few considering the high frequency of cysts present (please refer figure three of reference [1]). Also in adult testis the frequency of labeling was only 1.5% on an average (please refer Table one and Supplementary Figure one of reference [1]). Hence considering the very low frequency of labeling process, we think technical reasons might have contributed to failure of detection of germ cell cysts rather than absence of ovarian stem cells in adult ovary *per se*. We were even more surprised that the group expected stem cells to become functional after 4–14 days of busulphan treatment. It has been suggested that busulphan treatment destroys the somatic microenvironment of both testes and ovaries in addition to the germ cells [18,19]. A better approach would have been to wait for a month after busulphan treatment for complete loss of germ cells/follicles and then stimulate the ovary to study stem cell activity. Spontaneous stem cell activity is not likely to occur immediately after chemotherapy.

Similarly, another group also demonstrated lack of mitotically active female germ cells using alternative lineage tracing approach, using MVH as a marker [20]. We suggest technical limitations may exist in this study too and thus may have resulted in negative results. The important issue that needs to be resolved in this paper [20] is whether recombination by Cre occurs in ovarian stem cells in spite of them expressing MVH? In the original report that generated germ cell specific Vasa-Cre transgenic mice [21] it was reported that Vasa Cre activity was detectable only from e15-e18 in mouse testis even though Vasa expression in mice is reported as early as e11.5. The period of e15-e18 corresponds to meiosis and oocyte commitment and lack of mitosis in fetal ovaries. It was speculated that absence of remote or far away located regulatory elements might explain lack of earlier Cre activity. Another reason postulated was requirement of a threshold level of Cre protein to effectively induce recombination that was available only during the window period of e15-18 and thus enabled visual detection by Lac Z expression of Cre recombinase by X-gal method of detection and not earlier in spite of expression of MVH. This reason seems more likely because independent studies have shown that the levels of MVH increase by 2–2.5 fold in germ cells around e14.5 and e15.5 compared to e12.5 in both male and female mouse gonads [22,23]. Also, in a previous report by Araki et al. [24] it has been shown that in ES cells the efficiency of Cre mediated recombination depends on strength of promoter activity under-which it is cloned suggesting the need for appropriate levels of Cre protein for effective recombination. Hence the need for a threshold level of Cre protein to effectively induce recombination and subsequent reporter expression becomes important in the context of conclusions made recently [20]. The question that arises is whether the ovarian stem cells in adult mouse ovaries have strong enough MVH expression to induce sufficient Cre levels for recombination to occur? This seems unlikely since pre-meiotic mitotically active germ cells in fetal ovary do not exhibit Cre activity in spite of them expressing MVH (as these cells are observed prior to e15). Thus it is likely that even adult mitotically active ovarian stem cells and pre-meiotic germ cells may not exhibit Cre-induced recombination. Use of more primitive markers like OCT-4 or STELLA for lineage studies instead of MVH to conclusively determine the presence or absence of adult ovarian stem cells may be a better option. These markers are essential for oogenesis compared to MVH as knockout mice of these markers are known to be lethal or have led to complete loss of germ cells in females compared to MVH knockout, which does not affect female germ cells [25,26].

**Figure 2:**
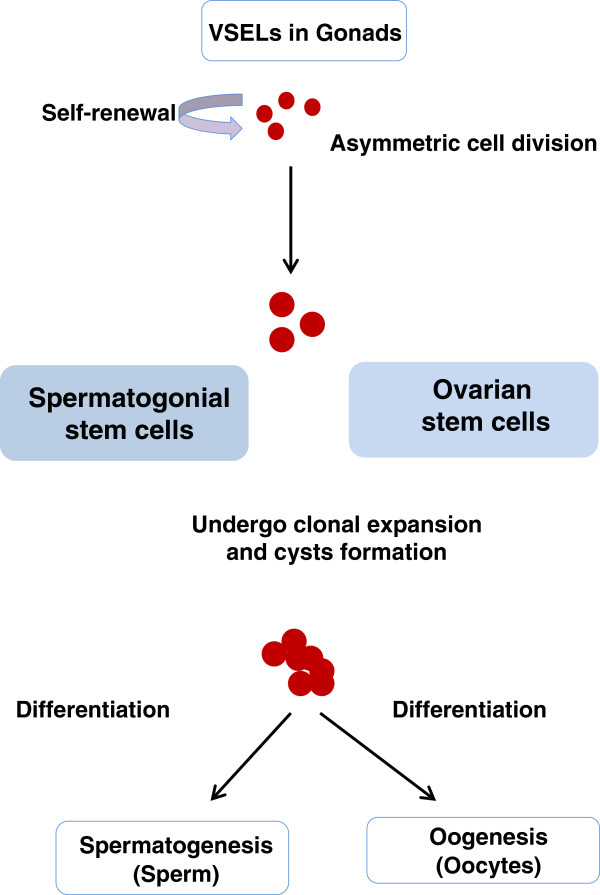
**Schematic representation of the presence of stem cells in the mammalian gonads.** VSELs are present in both ovary and testis. In the ovary the stem cells are lodged in the ovary surface epithelium whereas they are located in the basal epithelial layer in the testicular seminiferous tubules. VSELs (1–4 um) are relatively quiescent, undergo asymmetric cell division and give rise to the progenitors (5-10 um) which divide rapidly, undergo clonal expansion and symmetric cell divisions to form cysts and eventually differentiate into oocytes and sperm respectively in the ovary and testis. Presence of VSELs in the gonads needs to be acknowledged in addition to the recent understanding of gonadal stem cell biology proposed by Woods and Tilly [27].

We have shown that indeed both ovaries and testes harbor two stem cell populations including VSELs and the progenitors which are spermatogonial stem cells (SSCs) in testes and ovarian germ stem cells (OGSCs) in ovaries [7,13]. The OGSCs described by us are similar to the OSCs described recently by Jonathan Tilly’s group [27]. Tilly’s group has reported the presence of progenitors in mouse ovaries ranging in size from 5–8 um whereas we have reported an additional population of pluripotent, very small ES-like stem cells in adult mammalian gonads (Figure 2) which are possibly the primordial germ cells that persist into adulthood [28,29]. These cells exist and can be easily visualized in scraped OSE and testicular smears in contrast to recent doubts casted on the very presence of VSELs in adult tissues [30].

Based on the results and discussions, it is concluded that the findings of the two studies published in PNAS [1,20] should be interpreted with caution since absence of evidence is not necessarily the evidence of absence. Also we throw new light on stem cell biology in both ovary and testis.

## Competing interests

The authors declare that they have no competing interests.

## Authors’ contribution

All authors read and approved the final manuscript.
